# Transplantation of human fetal biliary tree stem/progenitor cells into two patients with advanced liver cirrhosis

**DOI:** 10.1186/s12876-014-0204-z

**Published:** 2014-12-04

**Authors:** Vincenzo Cardinale, Guido Carpino, Raffaele Gentile, Chiara Napoletano, Hassan Rahimi, Antonio Franchitto, Rossella Semeraro, Marianna Nuti, Paolo Onori, Pasquale Bartolomeo Berloco, Massimo Rossi, Daniela Bosco, Roberto Brunelli, Alice Fraveto, Cristina Napoli, Alessia Torrice, Manuela Gatto, Rosanna Venere, Carlo Bastianelli, Camilla Aliberti, Filippo Maria Salvatori, Luciano Bresadola, Mario Bezzi, Adolfo Francesco Attili, Lola Reid, Eugenio Gaudio, Domenico Alvaro

**Affiliations:** Department of Medico-Surgical Sciences and Biotechnologies, Polo Pontino, Sapienza University of Rome, Corso della Repubblica 79, Latina, 04100 Italy; Department of Movement, Human and Health Sciences, Division of Health Sciences, University of Rome “Foro Italico”, Rome, 00151 Italy; Department of Experimental Medicine, Viale Regina Elena 324, Rome, 00161 Italy; Department of Anatomical, Histological, Forensic Medicine and Orthopedics Sciences, Via Alfonso Borelli 50, Rome, 00185 Italy; Eleonora Lorillard Spencer-Cenci Foundation, Rome, 00100 Italy; Department of General Surgery and Organ Transplantation, Viale del Policlinico 155, Rome, 00161 Italy; Department of Gynecologic-Obstetric and Urologic Sciences, Viale Regina Elena 324, Rome, 00161 Italy; Department of Radiological Sciences, Viale Regina Elena 324, Rome, 00161 Italy; Department of Clinical Medicine, Sapienza University of Rome, Rome, 00185 Italy; Department of Cell Biology and Physiology, Program in Molecular Biology and Biotechnology, UNC School of Medicine, Chapel Hill, NC 27599 USA

**Keywords:** Human biliary tree stem/progenitor cells, Cirrhosis, Stem cell therapy, Clinical trial

## Abstract

**Background:**

Efforts to identify cell sources and approaches for cell therapy of liver diseases are ongoing, taking into consideration the limits recognized for adult liver tissue and for other forms of stem cells. In the present study, we described the first procedure of *via* hepatic artery transplantation of human fetal biliary tree stem cells in patients with advanced cirrhosis.

**Methods:**

The cells were immune-sorted from human fetal biliary tree by protocols in accordance with current good manufacturing practice (cGMP) and extensively characterized. Two patients with advanced liver cirrhosis (Child-Pugh C) have been submitted to the procedure and observed through a 12 months follow-up.

**Results:**

The resulting procedure was found absolutely safe. Immuno-suppressants were not required, and the patients did not display any adverse effects correlated with cell transplantation or suggestive of immunological complications. From a clinical point of view, both patients showed biochemical and clinical improvement during the 6 month follow-up and the second patient maintained a stable improvement for 12 months.

**Conclusion:**

This report represents proof of the concept that the human fetal biliary tree stem cells are a suitable and large source for cell therapy of liver cirrhosis. The isolation procedure can be carried out under cGMP conditions and, finally, the infusion procedure is easy and safe for the patients. This represents the basis for forthcoming controlled clinical trials.

**Electronic supplementary material:**

The online version of this article (doi:10.1186/s12876-014-0204-z) contains supplementary material, which is available to authorized users.

## Background

Human biliary tree stem/progenitor cells (hBTSCs) have been isolated from fetal and adult large intrahepatic and extrahepatic bile ducts and found to reside within the peribiliary glands (PBGs) and crypts of gallbladder epithelium [1,2]. The hBTSCs were able to self-replicate in culture and to differentiate into mature hepatocytes, cholangiocyte, or pancreatic β-cells *in vitro* and i*n vivo* [3-5]. The aim of this study was to evaluate the safety and the feasibility of a therapeutic protocol for advanced liver cirrhosis based on transplantation of hBTSCs.

## Methods

### Human fetal livers

The study was approved by the local ethics committee of Sapienza University Hospital. The livers from human fetuses consisted of one of gestational age of 18 weeks and used in patient#1 (male), and one of gestational age of 20 weeks and used in patient#2 (female). They were obtained by elective pregnancy termination from the Department of Gynecology (Sapienza, University of Rome, Italy). Patients signed the donation consent after they have independently signed the consent to the abortion, and after the abortion has been initiated by the administration of the drug for the delivery induction.

Serologic negativity for infectious diseases (HCV, HBV, HIV, EBV, HEV, HDV, toxoplasmosis, rubella, cytomegalovirus, parvovirus, herpes simplex type 1 and 2, TPHA) was documented in the mother as required by current regulation. Both fetuses presented severe cardiac malformations but no chromosomal alterations as demonstrated by karyotype analysis. To avoid prolonged ischemia we performed a monitoring of the fetal cardiac beat every 3 hours during the abortion procedure. The fetal livers (fetus#1: 8 g weight; fetus#2: 12 g weight) including biliary tree and gallbladder were procured immediately after delivery and transported in refrigerated organ transport cases. A small fragment was taken for histology.

### Isolation of hBTSCs

The extrahepatic biliary tree (including the gallbladder) was maintained in position by pins at the hilum; the more peripheral liver parenchyma was detached from the biliary tree with a scraper. Successively, the entire biliary tree and the remaining parenchyma were further disaggregated gently by scalpel and a MACS dissociator (Miltenyi Biotec), and digested in buffer containing 300 U/ml type I Collagenase (Sigma Aldrich) and 0.3 mg/ml deoxyribonuclease (Sigma Aldrich) for 20–30 min at 37°C. Freshly isolated cells were immunoselected for EpCAM-positive cells using magnetic beads (Miltenyi biotec) [5,6]. The sorting resulted in isolation of 42 million viable cells from the first fetus and 60 million from the second one. The duration of the isolation procedure averaged 5 hours. Cells were suspended in sterile 10% glucose solution at 1 million cells *per* ml and maintained for 45 minutes under controlled temperature of 4°C before infusion. All the procedures were carried out according to “The rules governing medicinal products in the European Union” and the European guidelines of GMP for medicinal products for human use (EudraLex-Volume 4 Good manufacturing practice Guidelines). Cell products were evaluated by standard sterility tests for gram+, gram-, aerobic and anaerobic bacteria, mycetes and with endotoxins tests, and characterized immediately by Flow Cytometry (FC) for EpCAM (Miltenyi Biotec, CD326/EpCAM-FITC, human; dilution 1:50) and LGR5 (OriGene, LGR5-PE, human; dilution 1:50) before transplantation in the patients.

### Patients’ inclusion and exclusion criteria

The inclusion criteria were: 1. Child-Pugh class C (score = or >10), and 2. Patients who were not candidates for liver transplantation because of their age being greater than 65 years and because of surgical or anesthetic risk. 3. Informed consents were obtained from the patients undergoing to the cell infusion procedure. The exclusion criteria were: 1. Patients with contraindications to hepatic artery cannulation; 2. Chronic hepatic encephalopathy that renders unenforceable the request of informed consent; 3. Patients with portal vein thrombosis; 4. Active alcoholism; 5. Active infections; 6. Primary liver cancers or liver metastases; 6. Spontaneous Bacterial Peritonitis; 7. Hepato-renal syndrome. Written informed consent was obtained from the patients for publication of their clinical details and any accompanying images. A copies of the written consents are available for review by the Editor of this journal. The study was approved by our local Ethic Committee, Policlinico Umberto I/Sapienza University of Rome and was non-sponsored (protocol code, 1951).

Other methological details were furnished in Additional file 1.

## Results

### *In situ* characterization of EpCAM-positive cells in fetal biliary tree and liver

**Figure 1:**
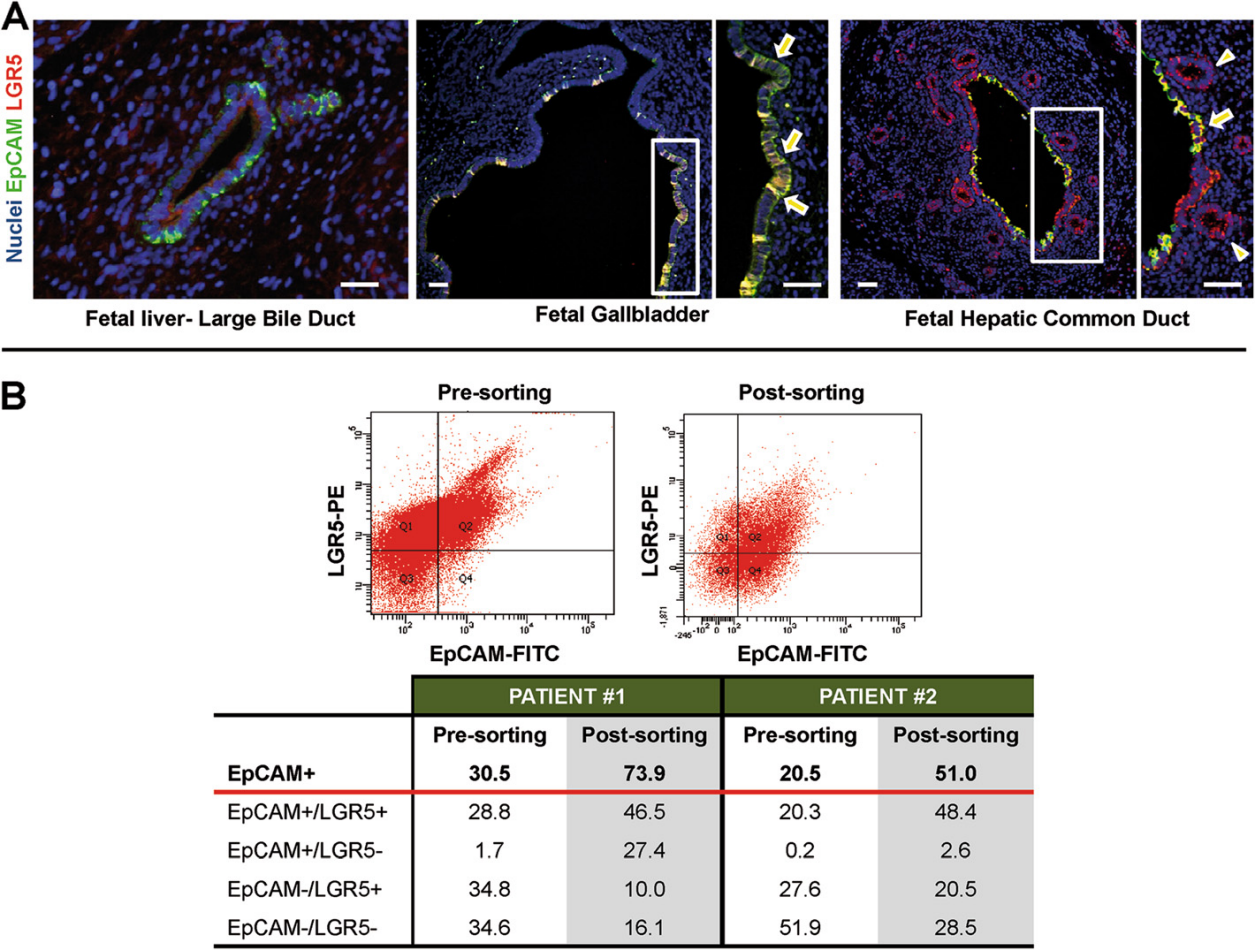
**Immunphenotype of biliary tree stem/progenitor cells and flow cytometry of transplated cells. A)** Expression of EpCAM and LGR5 in human fetal livers and extrahepatic biliary tree. Double immunofluorescence for EpCAM (green) and LGR5 (red) in human fetal liver. Larger intrahepatic bile ducts are EpCAM and LGR5 positive. scale bar = 50 μm. Epithelial cells of gallbladder are EpCAM and LGR5 positive (arrows). scale bar = 50 μm. Cells in surface epithelium are EpCAM and LGR5 positive (arrows). Cells in peribilary glands are mostly EpCAM-negative and LGR5-positive (arrowheads). scale bar = 50 μm. **B)** Flow cytometry plot for EpCAM and LGR5 of cell suspension prepared for patient #1. FC analysis of cell suspensions prepared for patient #1 and #2.

In fetal tissues, EpCAM was expressed by cells at different anatomical portions of the intrahepatic and extrahepatic biliary tree (Figure 1). Interestingly, most EpCAM-positive cells co-expressed LGR5. In fetal livers, LGR5-positive cells were located in the ductal plate and in the epithelium of larger bile ducts. In gallbladder and hepatic common duct, surface epithelial cells and bud of PBGs were diffusely positive for LGR5.

### Control tests and characterization of cell product

Estimated cell viability by trypan blue exclusion was routinely higher than 95%. All the microbiology tests resulted negative. The FC analyses of the first sample before EpCAM sorting, indicated that 30.5% of the freshly isolated cells were EpCAM-positive (Figure 1). The immunomagnetic sorting enriched for cells with co-expression of EpCAM and LGR5 (46.5%). A restricted sub-population was composed of EpCAM-negative but LGR5-positive cells (10.0%). The FC analyses of the second sample showed that, before sorting, 20.5% of the freshly isolated cells were EpCAM-positive. The immunomagnetic sorting enriched the EpCAM-positive population to 51% and contained cells with co-expression of EpCAM and LGR5 (48.4%). A significant sub-population was composed of EpCAM-negative but LGR5-positive cells (20.5%).

Immunosorted EpCAM-positive cells were suspended in 10% glucose solution, at a concentration of 1 million cells per ml. The cell suspension was infused into the hepatic artery at an infusion velocity of 200 ml/h. The first patient presented an anatomical variant of the origin of the common hepatic artery. He received, via the right hepatic artery, 42 millions viable EpCAM-sorted cells. The patient #2 received, via common hepatic artery, 60 millions viable EpCAM-sorted cells.

### Clinical outcome

The clinical and biochemical parameters of the two cirrhotic patients transplanted with hBTSCs are given in Table 1. The first patient was a 73-year Caucasian male affected by HCV-related liver cirrhosis. The patient was also affected by auto-immune hemolytic anemia diagnosed 3–4 years before treatment. Table 1 shows the time course of significant biochemical and clinical parameters during the follow-up. Interestingly, albumin demonstrated an evident increasing trend that parallels a gradual and constant decrease of INR. Before treatment, the patient received repeated hospitalizations, mostly for treatment of ascites trough large volume paracentesis but, their duration was significantly shortened by the cell therapy (1-day vs 5-days hospital stay pre-treatment). The need of paracentesis was significantly reduced during the follow-up, with no request after cell therapy. Compressively, the control of ascites and the disappearance of leg edema were associated with a weight loss of 6 kg. The patient observed the same pharmacological treatment during the whole period of observation, and did not received albumin injection. Total bilirubin showed a separate kinetic pattern with respect to the other hepatic tests being affected by auto-immune hemolytic anemia. Six months after receiving hBTSC transplantation (Table 1) the Child-Pugh score decreased from C-12 to C-10 and, MELD score from 24 to 20, mainly due to improvement of coagulation and ascites. In the second semester of observation the patients displayed a gradual but constant worsening of liver functions (Table 1) with Child-Pugh’s and MELD scores returning to pretreatment values. However, in the same period, the patient underwent a femoral neck fracture complicated by pneumonia and died on October 2013 for pulmonary edema. With respect to the second patient, she is a Caucasian female patient affected by HCV-related cirrhosis. No side-effects related to the cell infusion were registered during the scheduled 6-month follow-up. Six months after the treatment (Table 1), the patient displayed a consistent amelioration of liver function: Child-Pugh score from C-11 to B-8, MELD score from 21 to 16. As illustrated in Table 1, after 12^th^ month of follow-up the patient continued to maintain a state of compensated liver cirrhosis. Notably, patient #2 experienced a gradual and constant amelioration of albumin value and coagulation (INR) along the 12 months follow-up as showed in the Table 1. The patient did not receive intravenous albumin infusion.

**Table 1:** **Clinical and biochemical parameters during the follow-up**

**Patient #1 Male, 73 ys/old**	**Baseline**	**2** ^**nd**^ **month**	**3** ^**rd**^ **month**	**6** ^**th**^ **month**	**12** ^**th**^ **month**
**Child-Pugh score**	12	10	11	10	12
**MELD score**	24	21	21	20	25
**Bilirubin (mg/dl)**	12.06	10.37	9.06	10.19	14.96
**Albumin (g/dl)**	3.0	3.3	3.04	3.18	3.2
**INR**	2.07	1.69	1.77	1.57	2.1
**Creatinine (mg/dl)**	0.6	0.6	0.7	0.7	0.8
ᅟ					
**Patient #2 Female, 71 ys/old**	**Baseline**	**2** ^**nd**^ **month**	**3** ^**rd**^ **month**	**6** ^**th**^ **month**	**12** ^**th**^ **month**
**Child-Pugh score**	11	10	9	8	8
**MELD score**	21	17	19	16	16
**Bilirubin (mg/dl)**	3.04	2.38	2.80	2.80	2.88
**Albumin (g/dl)**	3.2	3.24	3.69	3.80	4.00
**INR**	2.00	1.82	1.90	1.60	1.60
**Creatinine (mg/dl)**	1.30	1.10	1.20	1.08	1.07

## Discussion

Our proposal overcomes limitations of hepatocyte-based therapy [7,8] given the large availability of fetal tissues from therapeutic abortions and of adult biliary tree tissue typically discarded in liver and pancreatic transplantation procedures. Moreover, our cell isolation procedure represents a further advance since Khan et al. [9] did not include extrahepatic biliary tree or gallbladder that are tissues enriched in EpCAM/LGR5 positive cells. As far as cell product characterization, FACS analysis allowed us to evaluate the phenotype of the companion cells after cell sorting. They consisted of cells of mesenchymal origin (white blood, hematopoietic, stromal and endothelial cells) [4-6,10,11]. This apparent contamination is due to the fact that in the cases of solid organs the digestion procedure does not allow complete destruction of the cell-cell interactions [6]. The purity of the cell product could be further increased by culture strategy [4,5,10]. As far as clinical outcomes, during the whole period of the strict follow-up we did not observed any increase in necrosis indexes nor signs of rejection such as LDH, transaminases, inflammatory indexes (reactive C protein, sedimetry, leucocyte count). Here, the absence of signs of rejection and/or allergy without any immune-suppressive regimen, correlates with minimal or null expression of HLA class I and II antigens both in hepatic and biliary tree stem cells from fetal liver [5,6,9,10]. We have recently confirmed these data in our setting, showing how EpCAM-positive hBTSCs, residing in ductal plates and along the entire biliary tree, strongly express FAS ligand [10]. In this study, in *in vitro* experiments on hBTSCs co-cultured with T lymphocytes, FAS-mediated apoptosis of CD4 and CD8 T cells was induced [10]. EpCAM- sorted cells from fetal livers have been used already in clinical trials of cell therapy of advanced liver cirrhosis without the need of immune-suppressants [6,9]. Here, from a clinical point of view, both patients showed biochemical and clinical improvement during the 6 month follow-up and the second patient maintained a stable improvement for 12 months. Thus the 6^th^ month should result the more appropriate for a re-treatment. The lack of cell tracking in our report reduces its scientific value. However, using the same infusion route, Khan et al. [9] demonstrated, by a scintigraphic method, the effective engraftment in the liver.

## Conclusions

Our report represents the basis for protocols of future clinical trials. First of all, we employed minimal invasiveness in the procedure. These aspects have been demonstrated in this report as the patients were 71 and 74 years old and with advanced liver cirrhosis. The entire cell production was carried out in a certified cell factory accomplishing all the “European guidelines of GMP for medicinal products for human use”. Finally, an identity test was performed before the cell injection. Moreover, we performed a monitoring of the fetal cardiac beat every 3 hours during the abortion procedure, and processed tissues experienced less than 3 hours of in uterus cardiac beat absence.

## Additional file

Additional file 1: **Supplementary Material.**

## Abbreviations

hBTSC: Human biliary tree stem/progenitor cell
EBV: Epstein barr virus
EpCAM: Epithelial cell adhesion molecule
FACS: Fluorescence activated cell sorting
HBV: Hepatitis B virus
HCV: Hepatitis C virus
HDV: Hepatitis D virus
HEV: Hepatitis E virus
INR: International normalized ratio
LGR5: Leucine-rich repeat containing G protein-coupled receptor 5
MELD: Model for end-stage liver disease
TPHA: Treponema pallidum hemagglutination test
