# Supplementary material for: Transplantation of human fetal biliary tree stem/progenitor cells into two patients with advanced liver cirrhosis
Source: BMC Gastroenterol. 2014 Dec 4;14:204. doi: 10.1186/s12876-014-0204-z (PMC4267130; doi:10.1186/s12876-014-0204-z)
Supplement: Supplementary file 1 — Supplementary Material. [file 12876_2014_204_MOESM1_ESM.pdf]

SUPPLEMENTARY MATERIAL

Materials and Methods

Tissue Processing

Tissue specimens were processed as previously described [1-3]. In brief, tissues were digested in RPMI 1640 supplemented with 0.1% bovine serum albumin, 1nM selenium, antibiotics, type I collagenase (300 collagen digestion unit/ml), 0.3 mg/ml deoxyribonuclease, at 37°C with frequent agitation for 30-45 min. Suspensions were filtered through a 500 micron metallic mesh filter (IDEALE ACLRI9 inox stainless steel) and spin at 1200 RPM for 5 min before resuspension. Thereafter, cell suspensions were passed consecutively through 100 and 30 micron mesh filter; then, cell counting was carried-out by Fast-Read 102 (Biosigma Srl, Venice, Italy) and cell viability (expressed as % of total cells) by the Trypan Blue assay. Cell viability (trypan blue exclusion) was constantly higher than 95%. N° 4 aliquots, containing approximately 200,000 cells, were collected for flow cytometry.

EpCAM sorting procedure

Cells were sorted for EpCAM by using magnetic beads as indicated by the manufacturer (Miltenyi Biotec Inc., Germany). Briefly, the EpCAM+ cells were magnetically labeled with EpCAM MicroBeads (Miltenyi Biotec Inc., catalog #130-061-101). Then, the cell suspension was loaded onto a MACS LS Column (Miltenyi Biotec Inc., catalog #130-042-401) that was placed in the magnetic field of a MACS Separator. The magnetically labeled EpCAM+ cells were retained within the column while the unlabeled cells ran through. After removing the column from the magnetic field, the magnetically retained EpCAM+ cells were eluted as a positively selected cell fraction. The EpCAM+ cells were evaluated by cell count and cell viability as previous described. EpCAM+ cells were suspended in basal

medium at a concentration of 300,000 cells per ml, and used as the final cell suspension. N. 4 aliquots, containing approximately 200,000 cells, were collected for flow cytometry.

Cell Isolation in GMP conditions and sterility testing

To produce BTSCs in cGMP conditions for future clinical application, gallbladders were processed following “The rules governing medicinal products in the European Union" and the European guidelines of good manufacturing practices for medicinal products for human use (EudraLex - Volume 4 Good manufacturing practice Guidelines). Sterility testing was performed under cGMP conditions by a “direct inoculation method” at San Camillo/Forlanini Hospital and in accordance with guidelines of good manufacturing practices for medicinal products for human and veterinary use.

Light Microscopy (LM) and Immunofluorescence (IF)

Specimens were fixed in 10% buffered formalin for 2-4 hours, embedded in low-temperature-fusion paraffin (55-57°C), and 3-4 µm sections were stained with hematoxylin-eosin, according to standard protocols. For immunofluorescence, non-specific protein binding was blocked by 5% normal goat serum. Sections were incubated with first primary antibodies (EpCAM, Mouse IgG1, Dako Cytomation, code: M3525, dilution 1:50). Specimens were washed and incubated for 1h with labeled isotype-specific secondary antibodies (anti-mouse AlexaFluor-488, Invitrogen, Life Technologies Ltd, Paisley, UK); Then, specimens were incubated for 1h with a PE-conjugated anti-LGR5 antibody (Origene, code: TA400001, dilution: 1:50). Finally, specimens were counterstained with 4,6-diamidino-2-phenylindole (DAPI) for visualization of cell nuclei. For all immunoreactions, negative controls (the primary antibody was replaced with pre-immune serum) were also included. Sections/Cultures were examined in a coded fashion by Leica Microsystems DM 4500 B Light and Fluorescence Microscopy (Weltzlar, Germany)

equipped with a Jenoptik Prog Res C10 Plus Videocam (Jena, Germany). IF staining were also analyzed by Confocal Microscopy (Leica TCS-SP2). IF observations were processed with an Image Analysis System (IAS - Delta Sistemi, Roma- Italy) and were independently performed by two pathologists in a blind fashion.

Flow Cytometry (FC) Analysis.

Isolated cells were labeled with fluorescent primary antibodies or adequate isotype controls. Cells were resuspended at approximately 2 x 10^5 cells/ml in PBS. Primary antibodies included EpCAM (EpCAM-FITC, Miltenyi Biotec Inc., catalog #130-080-301), LGR5 (LGR5-PE, Origene Technologies Inc., Rockville, MD, USA catalog #TA400001). Cells were analyzed by a BD FACScanto^{tm} Flow Cytometer (Becton, Dickinson and Company, NJ, USA). Ten thousand events were acquired and analyzed by BD FACSDiva™ software (Becton, Dickinson and Company, NJ, USA).

REFERENCES

1. Schmelzer E, Zhang L, Bruce A, et al. Human hepatic stem cells from fetal and postnatal donors. J Exp Med 2007;204:1973-87.
2. Wang Y, Yao HL, Cui CB, et al. Paracrine signals from mesenchymal cell populations govern the expansion and differentiation of human hepatic stem cells to adult liver fates. Hepatology 2010;52:1443-54.
3. Wauthier E, Schmelzer E, Turner W, et al. Hepatic stem cells and hepatoblasts: identification, isolation, and ex vivo maintenance. Methods Cell Biol 2008;86:137-225.
